# Trafficking new GPCRs and regulators to primary cilia

**DOI:** 10.1186/2046-2530-1-S1-O32

**Published:** 2012-11-16

**Authors:** PK Jackson

**Affiliations:** 1 Genentech Inc., USA

## 

Genetic heterogeneity mirrors the clinical heterogeneity of JSRD. Over 17 genes have been identified, all encoding for proteins of the primary cilium, with intriguing allelic series and clinical correlations that have made these conditions a paradigmatic example of “splitting and lumping” in human genetics. Indeed, the same phenotype can be caused by mutations in distinct genes, and conversely the same gene can be responsible of distinct ciliopathies, such as JSRD, nephronophthisis, Senior-Loken, Bardet-Biedl and Meckel syndromes. Of note, there is clinical heterogeneity even within families, with affected siblings discordant for features such as encephalocele or retinal involvement. This extreme phenotypic variability associated with mutations in one and the same gene remains a main open question. An intriguing explanation has implicated an oligogenic model of inheritance (already demonstrated for Bardet-Biedl syndrome and other ciliopathies), in which mutations, rare variants and even polymorphisms at distinct loci epistatically interplay to modulate the ciliopathy phenotype. To date, mutation analyses of most JSRD-causative genes have not been performed in large cohorts, and the systematic genetic screening of multiple ciliopathy genes remains a still unmet need to address the complexity of JSRD genetics.

